# Estimation of the Total Parasite Biomass in Acute Falciparum Malaria from Plasma PfHRP2

**DOI:** 10.1371/journal.pmed.0020204

**Published:** 2005-08-23

**Authors:** Arjen M Dondorp, Varunee Desakorn, Wirichada Pongtavornpinyo, Duangjai Sahassananda, Kamolrat Silamut, Kesinee Chotivanich, Paul N Newton, Punnee Pitisuttithum, A. M Smithyman, Nicholas J White, Nicholas P. J Day

**Affiliations:** **1**Faculty of Tropical Medicine, Mahidol University, Bangkok, Thailand,; **2**Centre for Clinical Vaccinology and Tropical Medicine, Churchill Hospital, Oxford, United Kingdom,; **3**Wellcome Trust–Mahosot Hospital, Vientiane, Lao People's Democratic Republic,; **4**Cellabs, Brookvale, New South Wales, Australia; St. George's Hospital Medical SchoolUnited Kingdom

## Abstract

**Background:**

In falciparum malaria sequestration of erythrocytes containing mature forms of *Plasmodium falciparum* in the microvasculature of vital organs is central to pathology, but quantitation of this hidden sequestered parasite load in vivo has not previously been possible. The peripheral blood parasite count measures only the circulating, relatively non-pathogenic parasite numbers. *P. falciparum* releases a specific histidine-rich protein (PfHRP2) into plasma. Quantitative measurement of plasma PfHRP2 concentrations may reflect the total parasite biomass in falciparum malaria.

**Methods and Findings:**

We measured plasma concentrations of PfHRP2, using a quantitative antigen-capture enzyme-linked immunosorbent assay, in 337 adult patients with falciparum malaria of varying severity hospitalised on the Thai–Burmese border. Based on in vitro production rates, we constructed a model to link this measure to the total parasite burden in the patient. The estimated geometric mean parasite burden was 7 × 10^11^ (95% confidence interval [CI] 5.8 × 10^11^ to 8.5 × 10^11^) parasites per body, and was over six times higher in severe malaria (geometric mean 1.7 × 10^12^, 95% CI 1.3 × 10^12^ to 2.3 × 10^12^) than in patients hospitalised without signs of severity (geometric mean 2.8 × 10^11^, 95% CI 2.3 × 10^11^ to 3.5 × 10^11^; *p* < 0.001). Parasite burden was highest in patients who died (geometric mean 3.4 × 10^12^, 95% CI 1.9 × 10^12^ to 6.3 × 10^12^; *p* = 0.03). The calculated number of sequestered parasites increased with disease severity and was higher in patients with late developmental stages of *P. falciparum* present on peripheral blood smears. Comparing model and laboratory estimates of the time of sequestration suggested that admission to hospital with uncomplicated malaria often follows schizogony—but in severe malaria is unrelated to stage of parasite development.

**Conclusion:**

Plasma PfHRP2 concentrations may be used to estimate the total body parasite biomass in acute falciparum malaria. Severe malaria results from extensive sequestration of parasitised erythrocytes.

## Abstract 2

Measuring sequestered parasites using plasma PfHRP2 concentrations may provide a more accurate estimate of total parasite mass and hence severity in falciparum malaria.

## Introduction

Histidine-rich protein 2 (PfHRP2) is a 30-kDa protein produced by *Plasmodium falciparum* [1,2]. PfHRP2 is found in the parasite cytoplasm or parasite food vacuole, where it may play a role in the polymerization of heme to hemozoin [3,4]. It is also found in the host erythrocyte cytoplasm and red cell membrane [5]. PfHRP2 is released from infected erythrocytes as a water-soluble protein both in vivo and in vitro [6,7].

Sequestration of parasitised erythrocytes in the second half of the asexual life cycle (mature trophozoite and schizont stages) in the microvasculature of vital organs compromises the microcirculation, and is a central feature in the pathogenesis of falciparum malaria. Peripheral blood parasitaemia is very widely used to assess disease severity in malaria, but it is only a weak predictor of mortality in falciparum malaria, as the less pathogenic circulating stages can be counted whereas the more pathogenic sequestered mature parasitised erythrocytes are not seen and therefore not counted by the microscopist. These sequestered parasites secrete PfHRP2 into the plasma, and PfHRP2 is liberated at schizont rupture. The plasma concentration of this protein might therefore provide a better estimate for the patient's total parasite biomass, and hence be an accurate prognostic indicator. In a recent study we measured PfHRP2 quantitatively in synchronized *P. falciparum* cultures, and showed that approximately 89% of PfHRP2 is liberated at schizont rupture and that the variation in the amount released is limited [8]. In the current study we quantified plasma PfHRP2 concentrations in patients with falciparum malaria and, using a simple mathematical model, applied this approach to estimate the total body parasite biomass. We then related this to parameters of disease severity and outcome. Since the developmental stage distribution of circulating parasites also provides information on the sequestered parasites [9], this was also evaluated in relation to plasma PfHRP2 levels in these patients.

## Methods

### Patients

In total 170 patients with uncomplicated malaria and 167 patients with severe malaria who participated in antimalarial treatment studies in Sangklaburi Hospital in western Thailand and in Mae Sot Hospital in northwestern Thailand were included in the study. These are low, seasonal transmission areas. Severe disease is seen in patients of all ages. Disease severity was classified using a modification of definitions employed by Hien et al. [10]. Patients were classified as having severe malaria if they met one of the following criteria: (a) Glasgow Coma Score (GCS) < 11, (b) hematocrit (Hct) 100,000/μl, (c) jaundice with bilirubin > 42.8 μmol/l and with parasite count > 100,000/μl, (d) serum creatinine > 265 μmol/l with urine output < 400 ml/24 h, (e) hypoglycaemia with venous glucose < 2.2 mmol/l, (f) systolic blood pressure < 80 mm Hg with cool extremities, (g) peripheral asexual stage parasitaemia > 10%, (h) peripheral venous lactate > 4 mmol/l, or (i) peripheral venous bicarbonate < 15 mmol/l. Patients without any of these features were considered to have uncomplicated malaria, even when the clinical severity was still sufficient to warrant admission to hospital. Heparinised plasma samples were collected on admission from these patients and stored at −70 °C until PfHRP2 assay. A complete blood cell count was made at the same time, and blood films were stained with Field's stain for parasite count and staging. Mixed infections with other *Plasmodium* species were excluded. The stage of *P. falciparum* parasite development on blood films was assessed as described previously [11] and divided into seven different age groups (tiny rings 0–6 h, small rings 6–16 h, large rings 16–26 h, early trophozoites 26–30 h, middle trophozoites 30–34 h, late trophozoites 34–38 h, and schizonts 38–48 h) [11]. Basic biochemistry and plasma glucose and lactate levels were assessed on all admission samples. Patients were treated with oral artesunate and mefloquine for uncomplicated disease or with intravenous quinine or artesunate for severe malaria, and managed according to standard procedures [12,13]. All patients or their attendant relatives gave fully informed written consent to blood sampling. The patients were enrolled in a series of prospective studies approved by the ethical review subcommittee of the Thai Ministry of Public Health.

### PfHRP2 Enzyme-Linked Immunosorbent Assay

All samples were tested with the malaria Ag Celisa kits (Cellabs, Sydney, New South Wales, Australia) employing a sandwich ELISA. These kits utilize a monoclonal-antibody-based assay specific for *P. falciparum* and detect PfHRP2 antigen produced by the parasite. The assay was performed following the manufacturer's instruction. Briefly, microwells were precoated with anti–*P. falciparum* monoclonal capture antibody. Plasma samples in serial 2-fold dilutions together with positive and negative controls (100 μl) were added to the wells and incubated for 1 h at room temperature, followed by anti-falciparum antibody conjugate (diluted 1:200). Finally, tetramethyl benzidine substrate was added, and the sample was incubated for 15 min at room temperature. The reaction was then stopped by adding 2 M sulfuric acid, and the results were read spectrophotometrically at 450 nm in a Bio-Tek ELX 808 TM plate reader (Bio-Tek Instruments, Winooski, Vermont, United States). The cut-off level for defining a reading as positive was set at the absorbance value of the negative control plus 0.1 unit, as recommended by the manufacturer of the test kit. A positive reading for PfHRP2 antigen was defined as an absorbance value above the cut-off level. Purified PfHRP2 (kindly provided by D. Sullivan, John Hopkins School of Public Health) diluted in either normal human plasma or PBS-TWEEN at decreasing concentrations was also assayed to construct a titration curve and to determine the sensitivity of the test. Titration curves were similar for human plasma and PBS-TWEEN as diluents, and the titration curve was used to convert titres to actual concentrations of PfHRP2. The lowest detection limit of purified PfHRP2 was 1.5 ng/ml.

### Calculation of Total Body Parasite Biomass

In our previous study we showed that PfHRP2 is secreted in a stage-dependent manner by parasitised erythrocytes. In in vitro studies of four carefully synchronized cultures, the median (range) amount of PfHRP2 secreted per parasite per erythrocytic cycle was 5.2 × 10^−15^ g (1.1 × 10^−15^ to 13.0 × 10^−15^ g). A median of 89%, or 4.6 × 10^−15^ g, of the total PfHRP2 was liberated at schizont rupture; the remaining 11%, or 0.6 × 10^−15^ g, was secreted almost entirely in the second half of the 48-h erythrocytic cycle [8].

Since the in vivo plasma elimination kinetics of PfHRP2 affects the relationship between plasma PfHRP2 concentrations and parasite biomass, the kinetics of PfHRP2 was studied in 13 adult patients with falciparum malaria admitted in the Bangkok Hospital for Tropical Diseases, Bangkok, Thailand, ten of whom fulfilled the criteria for severe disease [10]. Two patients had cerebral malaria (GCS < 11), three were jaundiced (serum bilirubin > 42.8 μmol/l), and two had renal failure (serum creatinine > 265 μmol/l). Plasma PfHRP2 concentrations were measured on admission, and then on days 1, 3, 7, 14, 21, and 21 after admission. The data were analysed using the WinNonlin statistical package (Pharsight, Mountain View, California, United States). There was no evidence of concentration dependence, and a single exponent term gave the best fit to the decline in plasma PfHRP2 concentrations with time (i.e., elimination of PfHRP2 was a first-order process). The mean (standard deviation) plasma elimination half-life (*t*½) of PfHRP2 was 3.67 (1.47) d, or 1.84 erythrocytic cycles, and was not correlated with measures of disease severity or biochemical evidence of renal or hepatic dysfunction.

**Figure 1:**
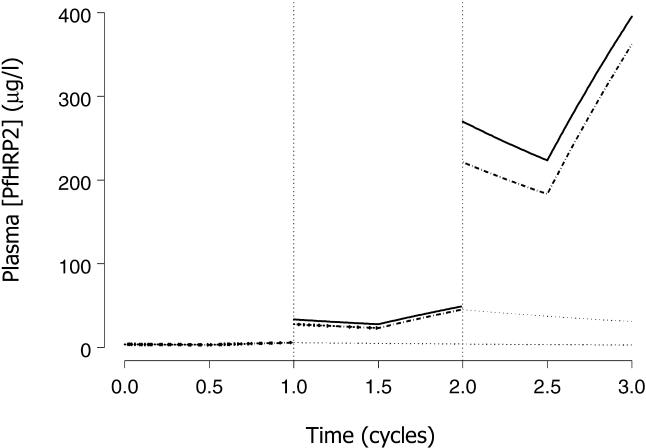
Model Describing the PfHRP2 Concentration Derived from 10^12^ Total Body Parasites in the Last Cycle Total PfHRP2 derives from the amount produced by the generation present in that cycle and the amount produced at schizont rupture of the previous cycle (broken lines). The concentration accumulated from previous production is added to this (black lines). PfHRP2 produced in previous cycles will decline over time (dotted lines). Values chosen for the parameters in this illustration were as follows: Hct, 0.35; body weight, 50 kg; and parasite multiplication factor, eight.

This information was used to construct a model to derive parasite numbers from the plasma PfHRP2 concentrations (Figure 1). It was assumed that patients were infected with a single relatively synchronous unimodal infection of *P. falciparum* [9]. Immediately following schizont rupture, released merozoites will infect new erythrocytes, expanding the infection with a multiplication factor *M*. If the number of parasites in the generation present at the moment of blood sampling is *P,* the number of parasites at the moment of the previous schizont rupture will be *P/M*. These parasites will release an amount of PfHRP2 (*A,* in grams) of 4.6 × 10^−15^ *P/M* into the circulation, giving a concentration of *A/V_d_,* where *V_d_* represents the volume of distribution of PfHRP2. As PfHRP2 is a relatively large molecule (30 kDa), it was assumed that *V_d_* equalled the plasma volume, which is (100 − Hct) × total blood volume. The total blood volume was estimated to be 80 ml/kg body weight.

As assessed in the pilot study described above, PfHRP2 elimination followed a first-order process, with a half-life (*t*½) of 3.67 d, or, for a 2-d cycle, 1.84 erythrocytic cycles, corresponding to an elimination constant *k* = 0.693/*t*½ = 0.377/asexual cycle. The concentration of PfHRP2 (in grams/litre) released at the moment of schizont rupture *(C_R_)* will then be given by the formula



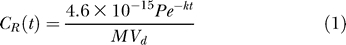



In the second half of the subsequent cycle, during the trophozoite and schizont stages, a total amount of 0.6 × 10^−15^ g of PfHRP2 per parasite will be released into the circulation by the newly infected erythrocytes. This production was assumed to be at a constant rate (1.2 × 10^−15^ g per parasite per cycle time) in the second half of the cycle. With time expressed as units of asexual cycle length and the elimination constant *k* = 0.377/cycle, the concentration *C_TS_* of PfHRP2 derived from these mature parasites during the second half of the erythocytic cycle will be given by the formula



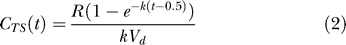



where *R* is the rate at which PfHRP2 is secreted into the plasma, equalling 1.2 × 10^−15^ *P* g per cycle, so that



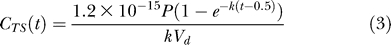



If *C*_0_ is the concentration of PfHRP2 derived from the current cycle, *C*_0_ *= C_R_* for the first half of the cycle, derived from schizont rupture of the previous cycle, and *C*_0_ = *C_R_* + *C_TS_* during the second half of the cycle, derived from the residual amount released at schizont rupture and from release by trophozoites and schizonts from the present cycle. Since all blood samples were taken on admission, even if the patient was admitted during the night, it was assumed that blood samples were drawn randomly during the erythrocytic cycle of the parasite. The average concentration of PfHRP2 (*C̄*_0_ ) will thus be the area under the concentration–time curve divided by the time span of the erythrocytic cycle of the parasite generation present at the moment of blood sampling. For one cycle from *t =* 0 until *t =* 1, this will equal



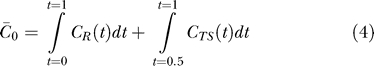



Substituting equations 1 and 3 into this equation, this can be solved to



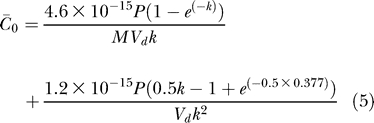



However, PfHRP2 accumulated from the erythrocytic cycles before the cycle during which the blood sample was taken will also contribute to the observed concentration of PfHRP2, because the elimination half-life exceeds the asexual cycle length. If *C̄*_1_ is the concentration of PfHRP2 derived from the cycle previous to the cycle at the moment of blood sampling, the amount of PfHRP2 (*C̄*_1_ ) produced in the previous cycle will be *C̄*_0_/*M* , and because of the exponential decline described by *k,* the average amount left in the next cycle will be *e* ^−*k*^(*C̄*_0_/*M*) .

For the *n* ^th^ previous cycle, the formula to calculate the contribution to the observed concentration of PfHRP2 can be generalized by induction to



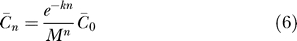



The total expected PfHRP2 concentration observed in a peripheral blood sample (*C̄*_obs_ ) can thus be expressed as



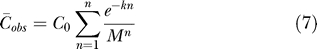



which can be solved to



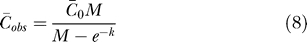



Substituting equation 8 into equation 5 provides an explicit expression for the overall concentration as a function of the parameters:



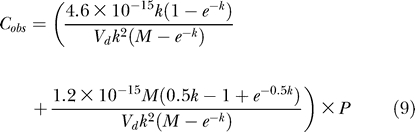



Figure 1 gives a graphic summary of the model.

Rearrangement of equation 9 gives an explicit expression for the total parasite load *P_tot_* as a function of the measured concentration:



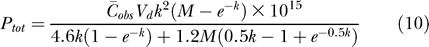



For the calculations of total body parasite biomass, an in vivo multiplication rate of eight was assumed. This is the average derived multiplication rate observed at detectable parasitaemias in patients with acute malaria in experimental infections before treatment [14]. Substituting these values into equation 10, and with the elimination constant *k* = 0.377, the total body number of parasites *(P_tot_)* can now be calculated for a measured plasma PfHRP2 concentration (*C_obs_*, in grams/litre):



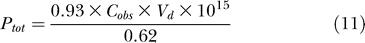



*V_d_* (in litres) was estimated as (1 − Hct) × 80 × 10^−3^ × body weight (in kilograms), equalling the estimated plasma volume, so that



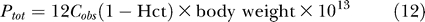



The total circulating number of parasites *(P_cir_)* was calculated from the observed peripheral blood parasitaemia (percent) in the peripheral blood thin smear multiplied by the estimated total body red blood cell mass. Assuming a total blood volume of 80 ml/kg body weight, total body red blood cell mass was estimated as



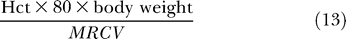



where *MRCV* is the mean red blood cell volume.

The estimated number of sequestered parasites was calculated as the difference between the estimated total parasite biomass and the total circulating parasite biomass.

The model was subjected to uncertainty and sensitivity analysis with the following parameters as variables: (a) multiplication rate *(M),* the distribution of which was chosen to be uniform between one and ten; (b) PfHRP2 half-life (*t*½), normally distributed with a mean of 3.7 d and a standard deviation of 1.47 d, derived from the pilot study described above; (c) the amount of PfHRP2 released per cycle, normally distributed with a mean of 5.2 × 10^−15^ g and a standard deviation of 2.0 × 10^−15^ g (from [8]). The fixed parameters included: Hct = 0.35% and body weight = 50 kg, giving a *V_d_* of 2.6 litres and an observed plasma PfHRP2 concentration of 1,000 × 10^−6^ g/l. Using these parameters 10,000 simulations were performed using the S-PLUS mathematical program (Insightful, Seattle, Washington, United States), with the predicted total parasite biomass as outcome. This showed that the predicted parasite biomass was log-normally distributed with a geometric mean of 2.9 × 10^12^. The coefficient of variation of the log-transformed outcome was 3%, indicating a high level of consistency.

**Figure 2:**
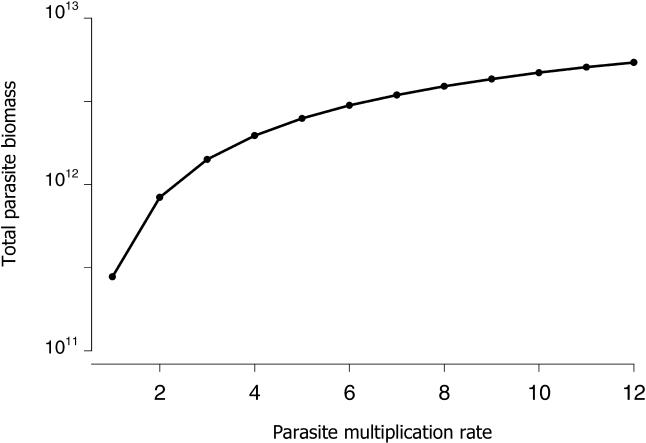
Estimated Total Parasite Biomass as a Function of the Parasite Multiplication Rate, Using the Model Described in the Text Values chosen for the parameters were as follows: PfHRP2 concentration, 1,000 μg/l; Hct, 0.35; and body weight, 50 kg.

The partial rank correlation coefficients between each parameter and the outcome of the model were 0.64, −0.10, and −0.48 for multiplication rate, PfHRP2 half-life, and the amount of PfHRP2 secreted per cycle, respectively. This indicates that multiplication rate was the most influential factor affecting the total parasite biomass estimate, followed by the amount of PfHRP2 secreted per cycle. The variation in PfHRP2 half-life had only a small effect on the calculations. To illustrate the impact of the multiplication rate on the model, Figure 2 shows the total body number of parasites for a patient with a plasma PfHRP2 concentration of 1 × 10^−3^ g/l, Hct of 0.35%, and a body weight of 50 kg at different parasite multiplication rates.

### Statistical Analysis

Data were analysed using SPSS for Windows release 10.05 (SPSS, Chicago, Illinois, United States). Continuous variables that were not normally distributed were log-transformed. The laboratory findings in the uncomplicated and severe groups were assessed using Student's *t*-tests for normal distributed parameters. Correlation was assessed by the methods of Pearson or Spearman as appropriate. Logistic regression was used to determine the relationship between mortality and plasma PfHRP2 or other prognostic factors including plasma haemoglobin, creatinine, lactate, bilirubin, and glucose concentrations.

## Results

### PfHRP2 Detection

The lowest limit of detection of purified PfHRP2 was 1.5 × 10^−6^ g/l. The titration curves were similar when normal human plasma or PBS-TWEEN was used as the diluent. The mean (95% CI) plasma PfHRP2 concentration in 337 patients with falciparum malaria was 8.4 × 10^−4^ g/l (5.7 × 10^−4^ to 11.1 × 10^−4^ g/l).

### Patient Characteristics

A total of 337 adult patients with falciparum malaria were enrolled over a period of 10 y. Patient characteristics are summarised in Table 1. According to the modified World Health Organization criteria adapted from Hien et al. [10], 167 patients had severe disease, of whom 28 (17%) died. Of the patients with severe disease 107 (64%) had cerebral malaria, defined as a GCS below 11. Twenty-two (13%) had renal failure (plasma creatinine > 265 μmol/l and diuresis < 400 ml/ 24 h), 87 (52%) were jaundiced (total plasma bilirubin > 42.8 μmol/l), and 73 (44%) had a plasma lactate above 5 mmol/l. Eight (5%) patients were shocked on admission (systolic blood pressure below 80 mm Hg, with cold extremities). No patient had an admission Hct below 20%. Seventy-two patients (43%) were hyperparasitaemic (>10% of red cells parasitised) as assessed in the peripheral blood slide on admission. Pre-treatment before admission with antimalarial drugs was infrequent and did not differ significantly between patients with severe versus uncomplicated disease. In the group with uncomplicated malaria (*n* = 170), three patients were treated with chloroquine (which is completely ineffective in this area), two received one dose of an artemisinin-containing drug, two received a single dose of quinine, and in two patients an unknown antimalarial was taken. For the patients with severe disease (*n* = 167), the corresponding numbers were as follows: four patients were pre-treated with chloroquine, one with sulphadoxine-pyrimethamine (also ineffective), two with a single dose of quinine, and in another six patients an unknown antimalarial was taken. So only seven out of 337 patients were documented to be treated with an effective antimalarial drug, and in none was the dose curative.

**Table 1:**
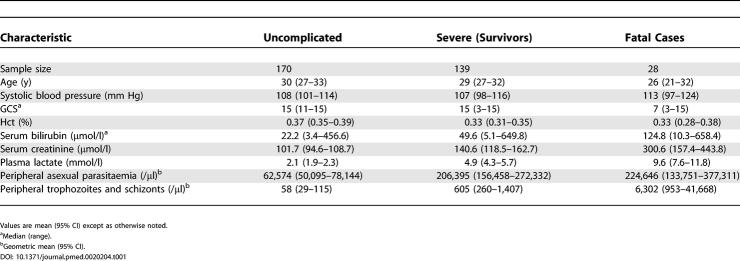
Characteristics of Studied Patients

### Estimated Total Body Parasite Biomass Compared to Peripheral Blood Parasitaemia and Parasite Developmental Stage

**Figure 3:**
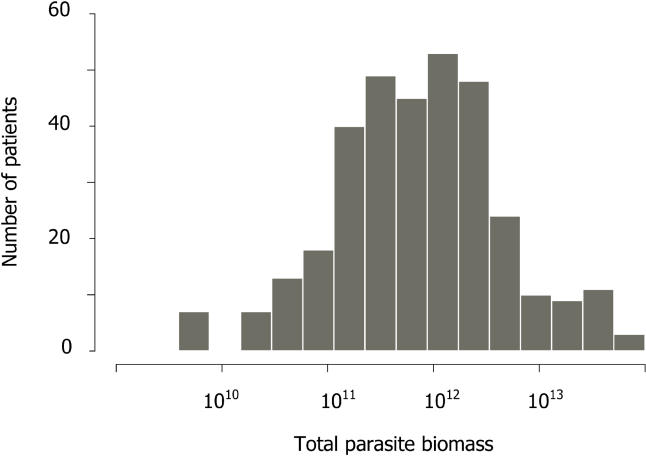
Total (Log_10_) Parasite Biomass Estimated from Plasma PfHRP2 Concentrations in a Peripheral Blood Sample in 337 Patients with Falciparum Malaria

Applying the model described above, the peripheral blood plasma concentration of PfHRP2 was used to estimate the total body parasite burden in 337 adult patients with falciparum malaria, assuming a uniform in vivo multiplication rate of eight (Figure 2). The geometric mean of the total parasite load for all patients was 7.0 × 10^11^ (95% CI 5.8 × 10^11^ to 8.5 × 10^11^) parasites/body (Figure 3). When the total parasite load was related to the estimated total red blood cell mass, the geometric mean whole body parasitaemia was 46 per 1,000 (95% CI 38 to 57 per 1,000) erythrocytes. The geometric mean of the simultaneously assessed peripheral blood parasitaemia for all patients was 28 per 1,000 (95% CI 25 to 33 per 1,000) erythrocytes.

When the numbers of sequestered parasites were calculated (by subtraction) a negative value was obtained in 22% of the patients; in these cases patients were assumed to have very few or no sequestered parasites. The median number of sequestered parasites obtained in this way for all malaria cases was 2.5 × 10^11^ (interquartile range 4.7 × 10^9^ to 1.5 × 10^12^) parasites/body.

The number of sequestered parasites was also assessed in relation to the parasite stage in the peripheral blood smear. We have reported previously that in patients with severe malaria a predominance of mature stages (trophozoites and schizonts) in the peripheral blood (>10^4^/μl) is associated with a fatal outcome, presumably because this reflects a stage distribution of more mature parasites composing the total parasite burden and thus a greater sequestered parasite biomass [9,11]. Using the previous cut-off value of >10^4^/μl mature-stage parasites in the peripheral blood, the number of sequestered parasites in patients with severe malaria (median [interquartile range]) was four times higher if mature-stage parastites predominated (*n* = 79); 1.6 × 10^12^ (2.5 × 10^11^ to 5.4 × 10^12^) parasites compared with 4.0 × 10^11^ (6.7 × 10^9^ to 2.1 × 10^12^) parasites if mature-stage parasites did not predominate (*p* = 0.002 by Mann-Whitney *U* test) (Figure S1). Overall there was a positive correlation between the proportion of late-stage parasites in the peripheral blood slide and the calculated proportion of sequestered parasites from the model (Spearman correlation, *r_s_* = 0.41, *p* < 0.001).

### Estimated Total Parasite Biomass and Severity of Disease

**Figure 4:**
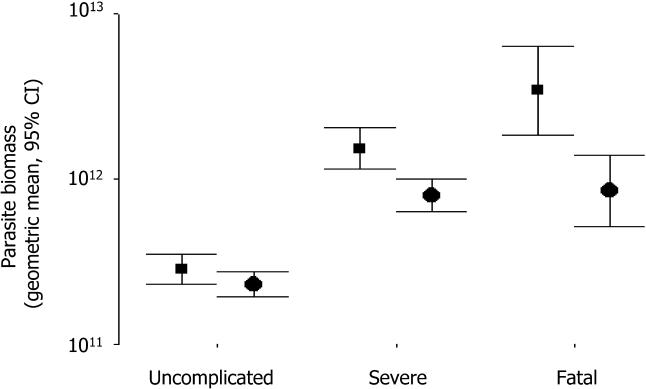
Total (Log_10_) Parasite Biomass Estimated from Plasma PfHRP2 Concentrations Plasma PfHRP2 concentrations (squares) in peripheral blood samples from 170 patients with uncomplicated malaria, 139 patients with severe malaria who survived, and 28 patients who died. For comparison the calculated circulating parasite biomass is also displayed (circles); this was calculated from the parasitaemia (per 1,000 red blood cells) in a peripheral blood sample and the estimated total red blood cell mass (see text for formulas).

The estimated total parasite biomass increased in proportion to severity of the disease.The geometric mean (95% CI) parasite load was six times lower in patients hospitalised without evidence of severe malaria than in patients with severe malaria: 2.8 × 10^11^ (2.3 × 10^11^ to 3.5 × 10^11^) versus 1.7 × 10^12^ (1.3 × 10^12^ to 2.3 × 10^12^) parasites/body (*p* < 0.001). Within the group of patients with severe malaria, those who died (*n* = 28) had over twice the geometric mean parasite load compared with patients who survived (*n* = 139): 3.4 × 10^12^ (95% CI 1.9 × 10^12^ to 6.3 × 10^12^) versus 1.5 × 10^12^ (95% CI 1.2 × 10^12^ to 2.0 × 10^12^) parasites/body (*p* = 0.03 with Bonferroni correction for multiple comparisons between the three groups; Figure 4). The calculated number of sequestered parasites also increased with severity of the disease. In “uncomplicated” malaria (*n* = 170) the median (interquartile range) number of sequestered parasites was ten times lower than in patients with severe malaria (*n* = 167): 9.0 × 10^10^ (6.7 × 10^9^ to 4.1 × 10^11^) compared to 9.1 × 10^11^ (6.1 × 10^10^ to 3.2 × 10^12^) parasites/body (Mann-Whitney *U* test, *p* < 0.001). Within the patients with severe disease, the median (interquartile range) calculated number of sequestered parasites was twice as high in the fatal cases than in the patients who recovered: 1.4 × 10^12^ (4.3 × 10^11^ to 1.4 × 10^13^) versus 7.6 × 10^11^ (1.2 × 10^10^ to 2.7 × 10^12^), but this difference was of borderline statistical significance (Mann-Whitney *U* test, *p* = 0.055).

### Estimated Total Parasite Biomass in Relation to Other Markers of Severity

Table 2 shows the associations between the estimated total parasite biomass and markers of disease severity. For comparison, the associations based on the calculated total circulating parasitaemia (from peripheral blood parasitaemia) with the same markers are shown. Within the 167 malaria patients with severe disease, 43 patients had cerebral malaria (defined as a GCS < 11 [10]). These patients had a higher estimated total parasite biomass than patients without coma, but also a higher peripheral blood parasitaemia. Plasma lactate concentrations can be regarded as a crude measure of the microcirculatory status and have a strong prognostic significance for mortality in severe malaria [15]. An admission plasma lactate concentration above 5 mmol/l indicates a poor prognosis. Patients with plasma lactate greater than 5 mmol/l (*n* = 73) had a significantly higher calculated total body parasite load than patients with a lower lactate concentration (*n* = 94): geometric mean (95% CI) 2.8 × 10^12^ (2.0 × 10^12^ to 4.1 × 10^12^) versus 1.3 × 10^12^ (0.9 × 10^12^ to 1.8 × 10^12^) parasites/body (Student's *t*-test, *p* = 0.002). The calculated sequestered parasite biomass was also significantly higher in patients with high plasma lactate concentrations (median 1.6 × 10^12^ parasites/body, interquartile range 9.4 × 10^10^ to 5.6 × 10^12^) than in those with an admission lactate concentration below 5 mmol/l (median 5.1 × 10^11^ parasites/body, interquartile range 9.1 × 10^9^ to 2.0 × 10^12^, Mann-Whitney *U* test, *p* = 0.012) (Figure S2). There was a positive correlation between the calculated sequestered biomass and the plasma lactate concentration on admission (Spearman correlation, *r_s_* = 0.44, *p* < 0.001). There was no association between the circulating parasitaemia (calculated from the observed peripheral parasitaemia) and plasma lactate concentration.

**Table 2:**
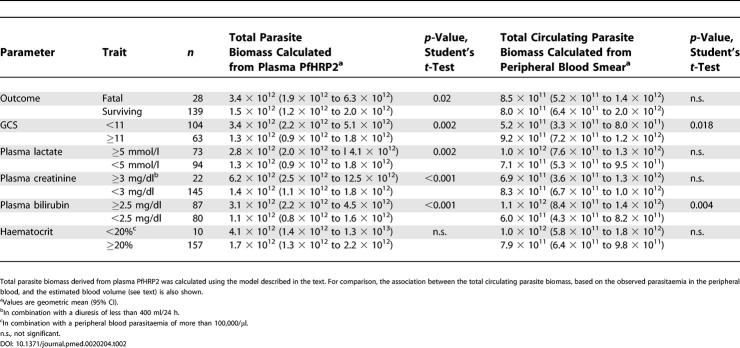
Association between the Estimated Total Parasite Biomass Derived from Plasma PfHRP2 and Established Measures of Disease Severity in Falciparum Malaria

Of the severe malaria patients, 22 had renal failure, defined as a plasma creatinine above 265 μmol/l and a diuresis of less than 400 ml/24 h [10]. These patients had a significantly higher estimated geometric mean total parasite load than the patients without renal failure. It should be noted that the plasma PfHRP2 half-life was not affected by renal failure in our pilot study. There was no association between peripheral blood parasitaemia and renal failure. Jaundiced patients (*n* = 87), defined as those having a total plasma bilirubin above 42.8 μmol/l [10], had significantly higher estimated total numbers of parasites, but also higher peripheral blood parasitaemias, than those without jaundice (*n* = 80). Finally, a slight but significant negative correlation existed between admission Hct and the calculated log_10_ total parasite biomass (Pearson's method, *n* = 167, *r* = −0.38, *p* < 0.001), but there was also a weak, though significant, correlation between Hct and the log_10_ total circulating number of parasites (*n* = 167, *r* = −0.20, *p* = 0.008). The group of severe anaemic patients as a whole did not have a significantly higher number of either circulating or total parasites in their blood (Table 2).

A logistic regression model was constructed with survival as the dependent variable and with the estimated total parasite biomass and previously described prognostic criteria in severe disease as independent variables [10]. Admission plasma lactate, plasma creatinine, and GCS contributed significantly in the model, but estimated total parasite biomass did not. This is in contrast to the univariate analysis, in which the estimated total parasite biomass and the sequestered parasite biomass (but not peripheral blood parasitaemia) correlated significantly with fatal outcome.

### Estimating the Time of Sequestration

It is known from laboratory studies that cytoadherence via the main adhesion molecule PfEMP1 begins at approximately 12 h of parasite development under febrile conditions, and 50% of the maximum effect is obtained at approximately 14–16 h of development [16]. This concurs with the stage distributions of parasites observed in the blood smears of patients with malaria.

If the time of admission to hospital and stage of parasite development (in hours) are unrelated, then the overall ratio of circulating parasites *(P_cir_)* to total parasites *(P_tot_)* in a population of patients gives the proportion of the asexual life cycle (assumed to be 48 h) for which parasites are circulating (*T_cir_,* in hours) at an overall parasite multiplication rate of one (i.e., steady state). That is,



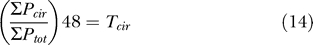



But the parasite numbers are usually expanding, particularly in uncomplicated malaria before treatment. In the present model we have assumed a multiplication rate of eight per cycle for preceding cycles. If the parasite age distribution is normally distributed with a standard deviation of 4 h, as estimated previously [9], then this provides 27% more circulating rings overall in the patient's parasite population than if the multiplication rate is one per cycle. That is,



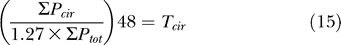



If applied to the data from the patients in this series and this model, then with the assumptions above, the population mean parasite age at sequestration *(T_cir_)* in uncomplicated malaria becomes



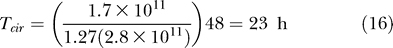



and for severe malaria is



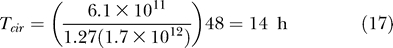



The estimate in severe malaria is very close to the value observed in the laboratory. But in severe malaria the next parasite cycle may not sustain a multiplication rate of eight. For a multiplication rate of one this value becomes 17 h. Values between 14 and 17 h are compatible with laboratory observations, but the estimate of 23 h for patients hospitalised with uncomplicated malaria is significantly different from the value observed in ex vivo studies. As it is very unlikely that the age of parasites when they sequester is truly different between the two groups, this apparent excess of circulating parasites in uncomplicated malaria suggests that the assumption of an admission to hospital unrelated to stage of parasite development is incorrect in this group. This suggests that schizogony and the consequent pathological reaction to it are important in stimulating patients with uncomplicated malaria to come to hospital, whereas referral of patients with severe malaria is unrelated to the stage of development of their infecting parasite population.

## Discussion

In this study we propose a model to estimate total parasite biomass using quantitation of a parasite-derived product (PfHRP2) that is predictably released into peripheral blood plasma in acute falciparum malaria. The estimated total parasite biomass was clearly associated with disease severity and outcome in univariate analyses. In a logistic regression analysis with outcome as dependent variable, the parasite load estimates did not predict disease severity better than conventional measures:GCS, plasma lactate, and serum creatinine outweighed the contribution of parasite load to the regression model, suggesting a close causal relationship between these variables. The estimated total parasite biomass was also associated with other well-established markers of severity. In contrast with this, peripheral blood parasitaemia and the derived total number of circulating parasites were not associated with disease outcome, nor with other important measures of severity such as admission plasma lactate concentrations. It should be noted that some of the criteria defining severe disease do include the level of the peripheral blood parasitaemia, which is variably related to total biomass, although in the model peripheral parasitaemia was not used in the calculations of parasite biomass. Pre-treatment with antimalarial drugs affecting PfHRP2 production cannot explain the differences in the estimated total parasite biomass between patients with severe versus uncomplicated malaria: documented pre-treatment was relatively rare (only seven out of 337 patients were pre-treated with an effective antimalarial drug), and this did not differ between the groups.

The positive association between sequestered parasite biomass and disease severity fits with the widely accepted view that the sequestration of erythrocytes containing the mature forms of the parasite in the microvasculature of vital organs is the central pathological process in falciparum malaria. The greater the number of sequestered parasites, the more severe is the disease. The cytoadherence of parasitised erythrocytes to the endothelial lining of the microcirculation is very efficient. Mature forms of the parasite are rarely seen in the peripheral blood smear used to assess the patient, whereas they are central to pathology. Overrepresentation of late stages in the peripheral blood smear is a prognostic factor for fatal outcome, because it represents a larger sequestered parasite biomass [9,11]. Marchiafava and Bignami were the first to observe in their pathological studies of falciparum malaria that “malignancy coincides with an exceptionally abundant quantity of parasitic forms, a quantity much more abundant—where the cases terminate fatally—in the blood of the viscera than in the blood of the finger” [17]. Recent autopsy studies comparing parasite numbers in brain vessels with peripheral blood parasitaemia showed a median sequestration index (the ratio of parasitaemia in the capillaries and venules compared to that in the peripheral blood) of 40 (range 1.8–1,500) [18], and a recent electron microscopic study by Pongponratn et al. in patients with severe malaria showed a sequestration index of 50 in brain capillaries, but lower indices in other vital organs [19]. In our study the geometric mean ratio between the total body parasitaemia and the peripheral blood parasitaemia in fatal cases was 5.3 (95% CI 2.11 to 13.4) (data not shown). As this ratio represents the summed estimate of sequestration in the whole body, it supports the pathological observations of differential sequestration in the brain and other vital organs.

The calculation of the sequestered biomass also revealed some limitations of the model we used, since in 22% of patients a negative value was calculated. This is not unexpected given that estimates for total parasite biomass and circulating parasite biomass derive from completely different parameters, and also that infections can be very synchronous [9]. Several factors may contribute to inaccuracies in the model. The amount of PfHRP2 secreted per parasite varies between different parasite strains; in our in vitro study the amount of PfHRP2 secreted per parasite per erythrocytic life cycle varied between 1.0 × 10^−15^ and 11.6 × 10^−15^ g. The antigenicity of PfHRP2 is not constant among parasite isolates because of variation in the expression of antigenic motifs. The calculations also assume a parasite multiplication factor of eight, which was derived from observations in early studies with experimental infection of humans with *P. falciparum* as a treatment for syphilis [14,20,21]. The multiplication factor has a significant impact on the model, as shown in the sensitivity analysis (see Figure 2). With lower multiplication factors, as are likely at high parasite densities, the contribution of the previous cycle to the total amount of PfHRP2 will increase, and a similar plasma concentration of PfHRP2 will represent a lower number of parasites. Indeed, in our model using a multiplication factor of eight, a small number of patients (21 out of 337 patients) had a calculated total number of parasites exceeding the calculated total number of erythrocytes. However, if a multiplication factor of one is used in these patients, no patient has a total body parasitaemia exceeding 100% of the calculated total number of parasites. Variation in another empirically derived estimate, the plasma half-life of PfHRP2, also has an impact on the model, but in the sensitivity analysis the effects were limited. In our study on plasma PfHRP2 kinetics in 13 patients with falciparum malaria, the longest plasma half-life was 6.6 d. Doubling the plasma PfHRP2 half-life in our model with an assumed multiplication factor of eight reduces the calculated total parasite load by a factor of 0.55. In high transmission areas, where partial immunity against the disease develops, clearance of PfHRP2 might be increased in the presence of antibodies against the protein. The model would thus underestimate the parasite burden and might need to be adapted for use in countries in sub-Saharan Africa. Another assumption made is that the volume of distribution *(V_d_)* of the plasma PfHRP2 equals the plasma volume, since the size of the molecule will likely confine its presence to the intravascular compartment. However, binding to other proteins or cells could increase the apparent *V_d_,* whereas dehydration could reduce *V_d_* slightly. An increase or decrease in *V_d_* will cause the model to under- or overestimate, respectively, the calculated total parasite load. Another source of variation is the PfHRP2 quantification itself. Higher concentrations of PfHRP2 require larger dilutions, with greater consequent errors. This may have contributed to the anomaly in those patients for whom the calculated total body parasitaemia as a percentage of circulating erythrocytes exceeded 100%. Finally, the time of blood-taking relative to the parasite developmental stage will influence the calculated parasite biomass. For example, assuming a multiplication factor of eight, an equal number of parasites will be associated with almost double the PfHRP2 concentration if the sample is drawn just before schizont rupture compared with halfway through the cycle, and the calculated parasite biomass will be affected by the same factor. *P. falciparum* infections do tend to be synchronous [9], and variations in PfHRP2 concentrations, and thus in the estimated biomass, related to the timing of the sample in relation to the stage of parasite development would be more substantial in those patients with synchronous infections. But the model predictions also provide an answer to this. By comparing the model-predicted parasite age at sequestration with the laboratory-observed values for cytoadherence, it was evident that in severe malaria the model gave a very good prediction compared with laboratory findings (14–17 h versus 14–16 h). This suggested that admission to hospital was unrelated to the timing of schizogony in severe malaria. But in patients with uncomplicated malaria, circulating stages were overrepresented, which suggests that schizogony, and the host reaction to it, probably do precipitate hospital admission. This is also supported by the predominance of parasites of the young ring stage observed commonly in admission hospital blood smears.

Despite all these potential shortcomings, estimates of the total parasite load derived from the plasma PfHRP2 concentrations were within the range expected and correlated well with disease severity. In clinical studies of severe malaria, measurement of plasma PfHRP2 concentrations may be useful as a research tool to stratify patients by their parasite load.

In summary, this study shows that quantitative measurements of plasma PfHRP2 in patients with falciparum malaria can be used to estimate the total parasite biomass, a parameter pivotal in the pathophysiology of the disease. This calculated total parasite biomass is associated with clinical measures of the severity of the disease.

## Supporting Information

Figure S1 Correlation between Sequestered Parasite Biomass and Percentage of Late Stages Scatterplot showing the correlation between the sequestered parasite biomass (as a percentage of the total parasite biomass) and the percentage of late stages (trophozoites and schizonts) in an admission peripheral blood slide in 337 patients with falciparum malaria. The sequestered and total parasite biomass were derived from plasma PfHRP2 concentrations as discussed in the text (Spearman correlation, *r_s_* = 0.41, *p* < 0.001).
(25 KB DOC).

Figure S2 Correlation between PfHRP2-Derived Parasite Biomass and Plasma Lactate Concentrations Scatterplot showing the correlation between the PfHRP2-derived sequestered parasite biomass on admission and admission plasma lactate concentrations in 337 patients with falciparum malaria (Spearman correlation, *r_s_* = 0.44, *p* < 0.001).
(28 KB DOC).

**Patient Summary**

**Background**

Malaria is caused by parasites of the genus *Plasmodium;* *P. falciparum* causes the most severe disease. After a person is bitten by an infected mosquito, the parasites enter the person's red blood cells (erythrocytes), and then these infected red blood cells get stuck in small blood vessels, blocking the blood flow and causing many of the problems associated with malaria. The parasites multiply inside the red cells, which eventually split and release more parasites.

**Why Was This Study Done?**

It is possible under the microscope to see the number of parasites in the circulating red blood cells but hard to measure how many are inside red blood cells stuck in the small blood vessels at any one time. Knowing the number of parasites blocking up the blood vessels might give a better idea of how severe the disease a patient has. An indirect measure of the number of unseen parasites in the body is a protein, PfHRP2, which the parasites produce and which is released into the blood when the red blood cells split.

**What Did the Researchers Do and Find?**

They measured the amount of PfHRP2 in the blood of 337 adult patients with *P. falciparum* malaria and looked mathematically to see whether the PfHRP2 measurements could be linked to the total number of parasites each person had in their body. They found that people with severe malaria had high total numbers of parasites hidden in their red blood cells, as measured by the PfHRP2 levels.

**What Do These Findings Mean?**

Further work will need to be done to reproduce these findings in other groups of people with malaria. However, estimating the total number of parasites in this way may make it possible to predict more accurately the likely severity of a person's disease.

**Where Can I Get More Information Online?**

The World Health Organization has a set of Web pages on malaria:

http://www.who.int/topics/malaria/en/

MedlinePlus has an interactive tutorial on malaria: http://www.nlm.nih.gov/medlineplus/tutorials/malaria/htm/index.htm

## Abbreviations

CI: confidence interval
GCS: Glasgow Coma Score
Hct: hematocrit
PfHRP2: Histidine-rich protein 2
