# Correction: Estimation of the Total Parasite Biomass in Acute Falciparum Malaria from Plasma PfHRP2

**DOI:** 10.1371/journal.pmed.0020390

**Published:** 2005-10-25

**Authors:** Arjen M Dondorp, Varunee Desakorn, Wirichada Pongtavornpinyo, Duangjai Sahassananda, Kamolrat Silamut, Kesinee Chotivanich, Paul N Newton, Punnee Pitisuttithum, A. M Smithyman, Nicholas J White, Nicholas P. J Day

In *PLoS Medicine*, volume 2, issue 8, DOI: 10.1371/journal.pmed.0020204

There are two data errors in the Results, under “Patient Characteristics”: of the patients with severe disease, 47 patients (28%) had cerebral malaria, and ten patients (6%) had an admission Hct below 20%.

Likewise, under “Estimated Total Parasite Biomass in Relation to Other Markers of Severity,” the text should indicate that 47 patients had cerebral malaria, and in Table 2, the number of patients with GCS < 11 should be 47.

