# Supplementary material for: Estimation of the Total Parasite Biomass in Acute Falciparum Malaria from Plasma PfHRP2
Source: PLoS Med. 2005 Aug 23;2(8):e204. doi: 10.1371/journal.pmed.0020204 (PMC1188247; doi:10.1371/journal.pmed.0020204)
Supplement: Supplementary file 1 [file pmed.0020204.sg001.doc]

Figure S1.

Scatterplot showing the correlation between the percentage of late stages (trophozoites and schizonts) in an admission peripheral blood slide and the sequestered parasite biomass (as a percentage of the total parasite biomass) in 337 patients with falciparum malaria. The sequestered and total parasite biomass were derived from plasma PfHRP2 concentrations as discussed in the text (r_s_ =0.41, p<0.001, Spearman correlation).
