# Supplementary material for: Estimation of the Total Parasite Biomass in Acute Falciparum Malaria from Plasma PfHRP2
Source: PLoS Med. 2005 Aug 23;2(8):e204. doi: 10.1371/journal.pmed.0020204 (PMC1188247; doi:10.1371/journal.pmed.0020204)
Supplement: Supplementary file 2 [file pmed.0020204.sg002.doc]

Figure S2.

Scatterplot showing the correlation between the PfHRP2 derived sequestered parasite biomass on admission (log scale) and admission plasma lactate concentrations (r_s_=0.44, p<0.001, Spearman correlation).

10^10^

10^11^

10^12^

10^13^

10^14^
